# Effect of oral contraceptive consumption timing on substrate metabolism, cognition, and exercise performance in females: a randomised controlled trial

**DOI:** 10.1007/s00421-025-05733-1

**Published:** 2025-03-03

**Authors:** Dan Martin, Mel Bargh, Kyla Pennington

**Affiliations:** 1 School of Sport and Exercise Science, College of Health and Science, University of Lincoln, Brayford Pool Campus, Lincoln, LN6 7TS UK; 2 Department of Psychology, University of York, York, UK

**Keywords:** Endocrinology, Oestrogen, Progestin, Exercise, Cognitive function

## Abstract

**Purpose:**

The pharmacokinetic profile of oral contraceptives (OCs) results in an acute, transient increase in circulating synthetic reproductive hormones. This study aimed to assess the acute effects of OC ingestion on cognitive function, substrate metabolism and exercise performance.

**Methods:**

Sixteen combined OC users ingested either their OC or placebo (PLA) in a randomised, double-blind, crossover manner. After 60 min, participants completed tests of verbal memory and verbal fluency, followed by sub-maximal treadmill exercise for 6 min at 70% lactate threshold (LT) and 90% LT where respiratory exchange ratio (RER), carbohydrate oxidation, fat oxidation, heart rate (HR), rating of perceived exertion (RPE), felt arousal and feeling scale were recorded. Participants then completed an incremental ramp test to exhaustion to assess time to exhaustion (TTE) and peak oxygen uptake (VO_2_peak), before ingesting the pill corresponding to the opposing condition

**Results:**

Compared to PLA, the OC condition has a significantly lower RER, arousal and feeling scale and significantly higher verbal fluency score (all *P* < 0.05) with no differences in other variables (*P* > 0.05).

**Conclusion:**

Combined OC ingestion acutely affects substrate metabolism, affective responses to exercise and verbal fluency. The timing of OC ingestion should be considered in relation to aspects of physiological function.

## Introduction

Reproductive hormones have been shown to influence substrate utilisation at rest and during exercise (Devries et al. 2006), exercise performance (McNulty et al. 2020) and cognitive function (Luine 2014). Given that most women of reproductive age have used at least one form of hormonal contraception (87.5%; Daniels & Abma 2023) and athletic populations have a high prevalence (~ 50%) of hormonal contraceptive use (Martin et al. 2018), it is important to understand the effects of exogenous/synthetic hormones contained within hormonal contraceptives on outcomes of importance for female athletes, exercisers, and the general population.

The effect of female reproductive hormones on substrate metabolism is evidenced by greater fat oxidation in females compared to males (Devries et al. 2006; Zehnder et al. 2005), and fat oxidation fluctuating across the menstrual cycle with the highest values observed during the luteal phase (Hackney et al. 1994; Zderic et al. 2001). The administration of exogenous oestrogen to amenorrheic women has been shown to reduce carbohydrate oxidation (Ruby et al. 1997). Several studies have shown reduced carbohydrate oxidation, reduced blood glucose concentrations and increased growth hormone concentrations in combined OC users vs. non-users (Bemben et al. 1992; Bonen et al. 1991). Furthermore, Ortega-Santos et al. (2018) showed that fat oxidation during exercise was significantly higher during pill consumption compared to the pill-free interval, suggesting that the exogenous hormones contained within OCs may be beneficial for enhancing fat oxidation. However, not all findings support this (*e.g.,* Issaco et al. 2012) and research on the effects of the menstrual cycle and OC use on exercise performance is equivocal likely due to poor study quality and heterogenous research designs (Elliott-Sale et al. 2020; McNulty et al. 2020).

Oestrogen may influence cognition via a range of mechanisms including rapid changes to cholinergic, monoaminergic, GABAergic and glutaminergic neurons (Luine 2014). As such, oestrogen concentrations have also been positively associated with aspects of cognition such as verbal memory and verbal fluency (Warren et al. 2014), which are superior in women compared to men (Hirnstein et al. 2023; Voyer et al. 2021) and superior in phases of the menstrual cycle with elevated oestrogen concentrations (Luine 2014; Maki et al. 2002; Rosenberg & Park 2002; Solís-Ortiz & Corsi-Cabrera 2008) albeit often with small to moderate effect sizes (*e.g*., D = 0.42; Maki, et al. 2002). A systematic review (Warren et al. 2014) identified that verbal memory is the cognitive domain most consistently improved with OC use. Verbal memory performance was superior in combined OC users compared to non-users (e.g., Gogos 2013) and verbal memory and verbal fluency performance were superior on pill consumption days compared to the pill-free interval (Mordecai et al. 2008).

To date, research has primarily compared hormonal contraceptive users to non-users or compared across phases of the menstrual cycle or OC cycle to evaluate the effects of reproductive hormones, however, this does not consider the pharmacokinetic profile and pharmacodynamics of OCs. Combined OCs, the most prevalent type of OC used in athletic populations (Martin et al. 2018), display typical first-order pharmacokinetics, whereby peak blood levels of ethinyl oestradiol and progestins are observed 60–120 min following ingestion of OCs (Dibbelt et al. 1991; Westhoff et al. 2010) followed by an exponential elimination proportionate to the concentration, with near total elimination at trough concentration (24 h). As such, there is only a window of a few hours whereby concentrations of ethinyl oestradiol and progestins are significantly elevated post-ingestion. Ethinyl oestradiol has been shown to have a greater affinity for oestrogen receptors (ER-α and ER-β) than naturally occurring oestrogens (Dickson & Eisenfeld 1981; Jeyakumar et al. 2011) and synthetic oestrogens have potent physiological effects such as increasing the risk of venous thromboembolism (van Hylckama Vlieg et al. 2009) and breast cancer (Beaber et al. 2014), as well as reducing the risk of developing ovarian cancer (Iversen et al. 2018).

No research to date has explored the effect of this predictable, consistent and acute rise in exogenous hormones on aspects of physiology and cognitive function, potentially due to the ethical constraints of supplementing individual doses of OCs to non-users. The current study utilises a novel study design to circumvent these ethical constraints; by using a double-blind, placebo-controlled experiment whereby the timing of OC ingestion is manipulated in existing OC users to compare participants when they have peak and trough exogenous hormone concentrations circulating in their system (Kuhnz et al. (1992). Utilising this novel design, the aim of this study was to assess whether the ingestion of a combined OC acutely affects cognition, substrate metabolism and exercise performance, compared to the ingestion of a placebo. The primary hypotheses were that following OC ingestion, RER will be lower, and verbal memory and verbal fluency will be superior, compared to the placebo condition.

## Materials and methods

### Participants

Twenty physically active (≥ 150 min moderate or ≥ 75 min vigorous intensity activity per week) female participants from the United Kingdom volunteered to take part in the study between January 2020 and December 2023. Following the withdrawal of 4 participants due to scheduling issues, 16 participants (age = 20.3 ± 2.3 y; height = 1.67 ± 0.08 m; body mass = 64.9 ± 11.2 kg) completed the study. An a priori sample size calculation using data from Hackney et al. (1994) identified that 12 participants are required for a power of β = 0.95, with respiratory exchange ratio (RER) as the main outcome measure and a sample size of 16 participants was needed for a power of β = 0.95 for verbal memory (Mordecai et al. 2008), therefore, the trial was stopped upon completion of 16 participants. Participants were required to have used a combined oral contraceptive with 30 µg ethinyl oestradiol and 150 mg Levonorgestrel (Rigevidon®, *n* = 12; Microgynon®, *n* = 2; Maexeni® 30, *n* = 1; Levest®, *n* = 1) for at least 6 months prior to participation, with a regimen of 21 pill consumption days and a 7-day pill-free interval. Further inclusion criteria were being aged 18–35 years, physically active, and speak fluent English. Exclusion criteria included polycystic ovarian syndrome, endometriosis, pregnancy, childbirth or lactation in the previous 6 months, BMI < 18.5 or > 30 kg·m^2^, smokers, any disorder known to affect metabolic health, history of head injury/neurological disorders and history of psychiatric disorders such as major depression or anxiety disorder. Ethical approval was received from the University of Lincoln Research Ethics Committee (718) and conformed to the standards of the Declaration of Helsinki. Participants were provided with a participant information sheet, completed a health screen, and gave their written informed consent in writing prior to commencing the study. Participants could withdraw from the study at any time.

### Experimental design

Participants completed a familiarisation session, followed by two main experimental trials in counterbalanced order in accordance with CONSORT guidelines (Schulz et al. 2010). Prior to the main trials, participants provided the technical team with two OCs from their pill packet to be allocated in a double-blind manner. The technical team conducted a simple randomisation (www.randomizer.org), ensuring allocation was independent of the research team. The allocation details were only revealed after the completion of data collection. Oral contraceptives and placebos (placebo-world.com; 80% lactose, 18.9% sucrose, 1.1% magnesium stearate) matched for colour, size and appearance were concealed in opaque cannisters with labels identifying allocation. In the OC main trial (OC), participants ingested their OC at 08:00 h, followed by the placebo at the completion of testing at approximately ~ 10:00 h. In the placebo trial (PLA), participants ingested the placebo at 08:00 h, followed by their OC after testing had been completed. As such, all participants consumed their prescribed OC during the duration of the testing session and did not miss a pill consumption day, with only the timing of ingestion being manipulated by approximately 2 h. A systematic review identified that missing one to four consecutive OCs during the pill consumption phase resulted in little follicular activity and a low risk of ovulation across 10 studies (Zapata et al. 2013). Furthermore, guidance contained within OC leaflets indicates that you are protected from pregnancy if one OC is 12 h late or less. Therefore, the proposed manipulation of OC timing is well within the bounds of acceptability and does not pose a risk of reduced contraceptive efficacy.

Main experimental trials took place on OC consumption days within the same OC cycle, separated by no more than 10 days to minimise the effect of synthetic hormone accretion over a pill consumption phase (Kuhnz et al. 1992). Testing did not take place during the first 3 days of pill consumption due to the elevated endogenous oestrogen concentrations at this time (van Heusden & Fauser 1999). Participants were asked to consume their OC at 08:00 on OC consumption days for at least 1 week before attending the laboratory for the main trials so that upon arrival to the laboratory they had not ingested their OC for 24 h. Participants were asked to avoid exercise and alcohol for 24 h before the main trials and to attend the laboratory following an overnight fast with only ad libitum water intake permitted.

### Experimental protocol

In the familiarisation session, participants completed a practice trial of the Rey Auditory Verbal Learning Test (RAVLT) and Verbal Fluency task using alternative task forms to reduce practice effects (Bell et al. 2018). Participants then completed a two-stage lactate threshold and VO_2_peak test (Jones 1998) to establish steady-state exercise intensities for the sub-maximal exercise assessment in the main trial and to familiarise participants with the maximal exercise test. Participants then completed a two-stage lactate threshold and VO_2_peak test (Jones 1998) to familiarise participants with the maximal exercise test and to determine steady-state exercise intensities for the sub-maximal exercise assessment in the main trials.

In the main trial, participants attended the laboratory at 08:00 and either ingested their OC or PLA in a double-blind manner according to the randomly generated sequence. Participants were asked to open the concealed container and ingest the tablet with water and with their eyes closed to reduce any possibility of them distinguishing between the OC and PLA conditions. Participants then rested for 60 min to allow time for the OC or PLA to enter the circulation, before completing the cognitive test battery.

### Cognitive tests

The cognitive test battery was performed in a quiet area, with participants seated at a desk facing a blank wall to minimise distractions. During the tests, the experimenter was seated approximately 1 m behind the participant and participants were instructed to face away from the experimenter throughout the tests.

Verbal memory was assessed using the RAVLT (Rey 1941) to assess immediate memory span, new learning, and susceptibility to interference. The RAVLT was chosen as previous research has shown superior performance during pill consumption days compared to the pill-free interval in a similar word recall task (Mordecai et al. 2008). A list of 15 words (List A) was read aloud for 5 consecutive trials (Trials 1–5), each followed by a free recall test during which participants were asked to recall as many words as possible from the word list. Following this, an interference list of 15 different words (List B) was read aloud, followed by a free recall of this list. Immediately after this, participants were asked to recall List A without further presentation of these words (Trial 6). After a 30-min delay, the participants were then asked to recall List A without hearing the words again (Trial 7). Time limits were not imposed, and participants were asked to inform the experimenter when they could not remember any more words. Acquisition (sum of all words), learning rate (difference between Trial 1 and 5), proactive interference (difference between words recalled in Trial 1 and List B), retroactive interference (difference between Trial 5 and 6) and forgetting (difference between Trial 7 and 5) were calculated. Alternate word lists with equivalent word length, serial position and frequency in the English language (Geffen et al. 1994) were counter-balanced across main trials.

The Verbal fluency test (Benton, 1989) is an assessment of the ability to produce words given certain restrictions and was chosen as this has been shown to be positively affected by oestrogen concentrations (Luine et al., 2014). In this test, participants were given a letter of the alphabet and were asked to say as many words as possible that begin with that letter in one minute, excluding capitalised words (*i.e.,* proper nouns) or variations of the same word (*e.g.,* garden and gardening). In each assessment, participants were given three different letters, with a short break between each. The total number of acceptable words said within the time limit was recorded with higher numbers equating to greater verbal fluency. The letter combinations used have been shown to have high equivalence (Cohen & Stanczak 2000) and the order of letter combinations was counter-balanced across main trials.

In addition to the above tests, a laptop-based cognitive testing battery was completed which included the mental rotation test (Vandenberg & Kuse 1978), Stroop-colour test (Stroop 1935), Corsi blocks test (Corsi 1972) and rapid visual information processing (RVIP) task (Wesnes & Warburton 1984). However, a technical issue resulted in data loss for 7 participants in these tests and, therefore, data are only available for 9 participants. We have not included these data in the paper as they are not sufficiently powered.

### Exercise tests

Immediately after the cognitive tests (~ 100 min post-OC/PLA ingestion), participants completed a sub-maximal exercise assessment on a treadmill (Mercury, h/p/cosmos, Germany) for 6 min at 70% (70%LT; 5.1 ± 0.9 km·h^−1^) and 6 min at 90% (90%LT; 6.6 ± 1.1 km·h^−1^) the treadmill speed corresponding to LT. Exercise intensities below LT were used as previous research has shown that during exercise at an intensity greater than LT, the demand for increased carbohydrate consumption surpasses the influence of oestrogen (Hackney 2021; Hackney et al. 1994). In the last minute of each stage, mean values for heart rate, breathing frequency and metabolic variables were collected using cardiopulmonary exercise test software (Metalyser 3B, Cortex, Germany). In the final 10 s of each stage, rating of perceived exertion (Borg 1970; scale 6–20), felt arousal (Svebak & Murgatroyd 1985; scale 1–6) and feeling scale (Hardy & Rejeski 1989; scale − 5 to + 5) were recorded by participants indicating a score using a laminated sheet. Respiratory exchange ratio (RER), carbohydrate oxidation and fat oxidation (Frayn 1983) and energy expenditure were calculated from the metabolic data. Immediately following the sub-maximal exercise test, participants completed a maximal treadmill ramp test at the same starting speed as the familiarisation, increasing the incline by 1% each minute until volitional exhaustion. Time to exhaustion, maximal heart rate and VO_2_peak were recorded. Following the completion of the exercise test, participants ingested the tablet (OC or PLA) that they had not ingested upon arrival, maintaining the double-blind design. No harms or unintended effects were reported for any participant.

### Statistical analysis

Heart rate, breathing frequency, RER, carbohydrate oxidation, fat oxidation, energy expenditure, RPE, arousal and feeling scale were compared between conditions (OC vs. PLA) and exercise intensities (70% vs. 90% LT) using two-way repeated measures ANOVAs, with Bonferroni correction. RAVLT outcomes, verbal fluency score, VO_2_peak, time to exhaustion and maximum HR during the ramp test were compared using paired-sample t-tests after checking for normality using Shapiro–Wilk tests. Data are presented as mean ± SD and statistical significance was set at *P* ≤ 0.05.

## Results

### Sub-maximal exercise

**Fig. 1:**
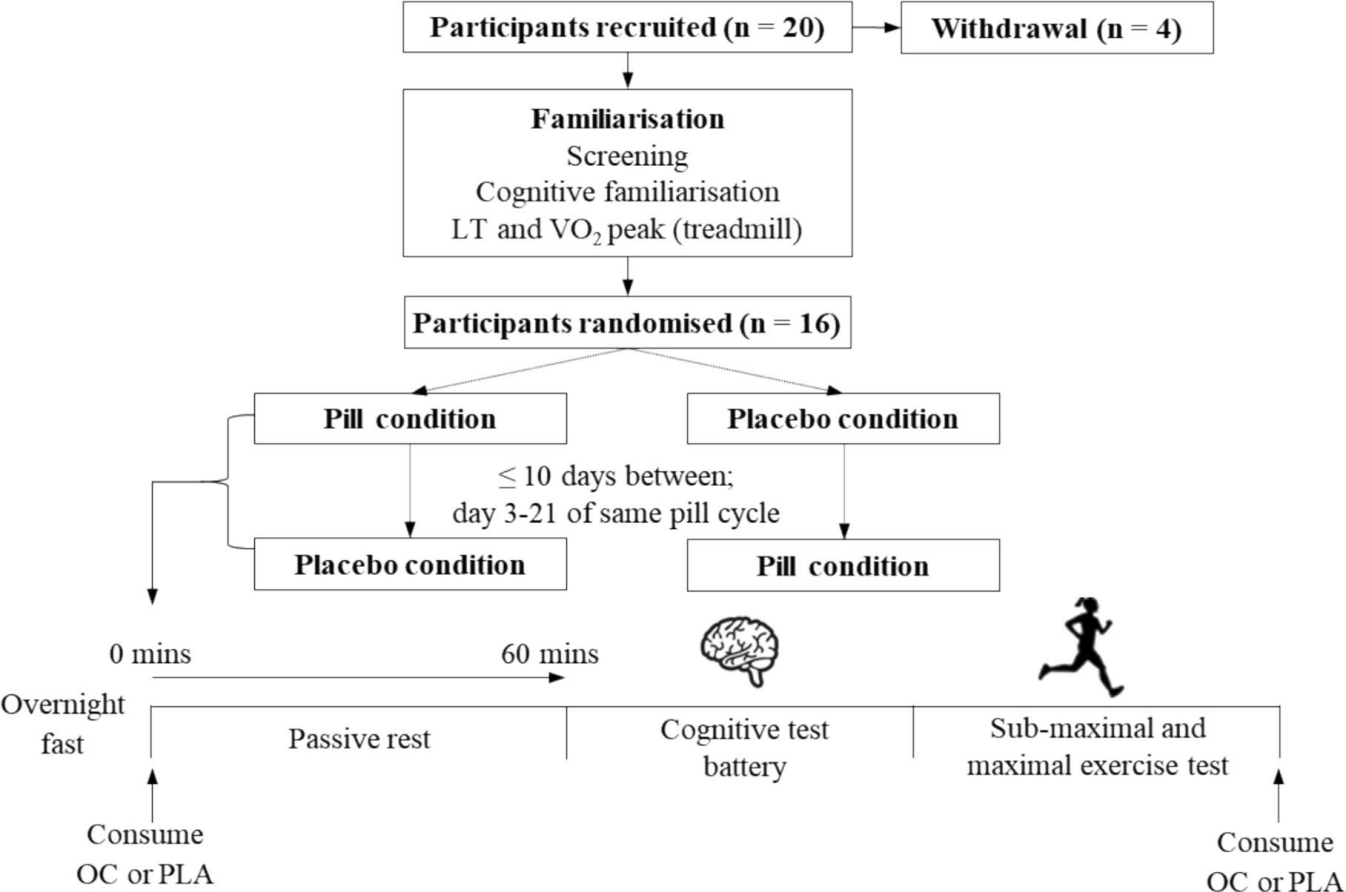
Schematic of the study. *LT* Lactate threshold, *VO*_*2*_*peak* Peak oxygen uptake, *OC* Oral contraceptive, *PLA* Placebo;

For RER, there was a significant effect of intensity (F(1, 15) = [151.68], *p* < 0.001, ηp^2^ = 0.910) and condition (F(1,15) = [7.90], *p* = 0.0132, ηp^2^ = 0.345) with no intensity x condition interaction (F(1,15) = [1.35], *p* = 0.263, ηp^2^ = 0.083). At 70%LT, RER was significantly lower in OC (0.855 ± 0.044) compared to PLA (0.871 ± 0.041, *p* = 0.0376, D = 0.37). AT 90%LT, RER was significantly lower in OC (0.917 ± 0.041) compared to PLA (0.939 ± 0.034, *p* = 0.0369, D = 0.59; Fig. 1). For heart rate, breathing frequency, carbohydrate oxidation, fat oxidation and energy expenditure there was a significant effect of intensity (all *p* < 0.05), with no condition or intensity x condition interaction (all > 0.05; Table 1).

**Table 1:** Mean ± SD measures during sub-maximal exercise for Oral Contraceptive (OC) and Placebo (PLA) conditions at 70% and 90% lactate threshold

	**70%**		**90%**	
	OC	PLA	OC	PLA
VO_2_ (L·min^−1^)	0.93 ± 0.17	0.96 ± 0.23	1.28 ± 0.31	1.29 ± 0.31
VCO_2_ (L·min^−1^)	0.80 ± 0.17	0.83 ± 0.20	1.17 ± 0.31	1.21 ± 0.30
Carbohydrate oxidation (g·min^−1^)	0.64 ± 0.28	0.70 ± 0.24	1.24 ± 0.45	1.35 ± 0.41
Fat oxidation (g·min^−1^)	0.22 ± 0.07	0.21 ± 0.09	0.17 ± 0.09	0.14 ± 0.11
Energy expenditure (kJ·min^−1^)	27.6 ± 34.9	19.7 ± 4.7	26.4 ± 6.5	27.00 ± 6.58
Breathing frequency (breaths·min^−1^)	26.6 ± 6.2	27.1 ± 6.4	33.9 ± 7.7	35.5 ± 7.5
Heart rate (beats·min^−1^)	113.3 ± 12.6	114.3 ± 13.4	142.4 ± 20.1	143.2 ± 19.6
RPE	8.4 ± 1.8	8.3 ± 1.7	10.1 ± 2.3	10.3 ± 2.0
Arousal	2.1 ± 1.1	2.6 ± 1.1 *	2.4 ± 1.1	2.6 ± 1.2
Feeling scale	1.8 ± 1.6	2.5 ± 1.5 *	1.6 ± 1.5	1.9 ± 1.6

*Indicates significant difference between OC and PLA

For arousal, there was a significant effect of condition (F(1,15), = [5.29], *p* = 0.0362, ηp^2^ = 0.261), with no significant effect of intensity (F(1, 15), = [1.52], *p* = 0.237, ηp^2^ = 0.092) or intensity x condition interaction (F(1,15), = [1.90], *p* = 0.188, ηp^2^ = 0.113). Post-hoc tests showed arousal was significantly lower (p = 0.0280, D = 0.39) in OC (2.13 ± 1.15) compared to PLA (2.56 ± 1.09) at 70%LT, with no significant difference at 90%LT (*p* > 0.05). For feeling scale, there was a significant effect of condition (F(1,15), = [8.23], *p* = 0.0117, ηp^2^ = 0.354), with no significant effect of intensity (F(1, 15), = [3.92], *p* = 0.0664, ηp^2^ = 0.207) or intensity x condition interaction (F(1,15), = [3.85], *p* = 0.07, ηp^2^ = 0.204). Post-hoc tests showed feeling was significantly (*p* = 0.026, D = 0.47) lower in OC (1.75 ± 1.61) compared to PLA (2.50 ± 1.55) at 70%LT, with no significant difference at 90%LT (*P* > 0.05). For RPE, there was a significant effect of intensity (F(1, 15), = [34.92], *p* < 0.001, ηp^2^ = 0.700), with no condition (F(1,15), = [0.03], *p* = 0.872, ηp^2^ = 0.002) or intensity x condition interaction (F(1,15), = [0.18], *p* = 0.676, ηp^2^ = 0.012; Table 1).

### Ramp test measures

There was no significant difference between OC and PLA for VO_2_peak (*P* = 0.0987; D = 0.44) time to exhaustion (*P* = 0.461, D = 0.189) or HR_max_ (*P* = 0.172, D = 0.357; Table 2).

**Table 2:** Mean ± SD ramp test measures

Measure	OC	PLA
VO_2_peak (L·min^−1^)	2.18 ± 0.334	2.13 ± 0.360
VO_2_peak (ml·kg·min^−1^)	33.9 ± 5.8	33.4 ± 5.6
TTE (s)	468.8 ± 78.1	463.9 ± 83.6
HR_max_ (beats·min^−1^)	196.2 ± 7.0	195.0 ± 7.4

### Verbal cognitive tests

There were no significant differences between OC and PLA conditions (all *P* > 0.5) for RAVLT acquisition, learning rate, proactive interference, retroactive interference and forgetting. Verbal fluency performance was significantly greater (*P* = 0.047; D = 0.54) in OC (45.9 ± 12. 7) compared to PLA (40.7 ± 8.3); Table 3).

**Table 3:** Mean ± SD cognitive test scores

Measure	OC	PLA
Acquisition	84.6 ± 15.0	85.9 ± 16.1
Learning rate	5.1 ± 2.1	5.3 ± 2.5
Proactive interference	1.3 ± 1.7	1.3 ± 2.5
Retroactive interference	1.7 ± 2.8	1.8 ± 1.4
Forgetting	2.1 ± 2.8	2.9 ± 2.3
Verbal fluency	45.9 ± 12.7	40.7 ± 8.3*

*Indicates significant difference between OC and PLA

## Discussion

**Fig. 2:**
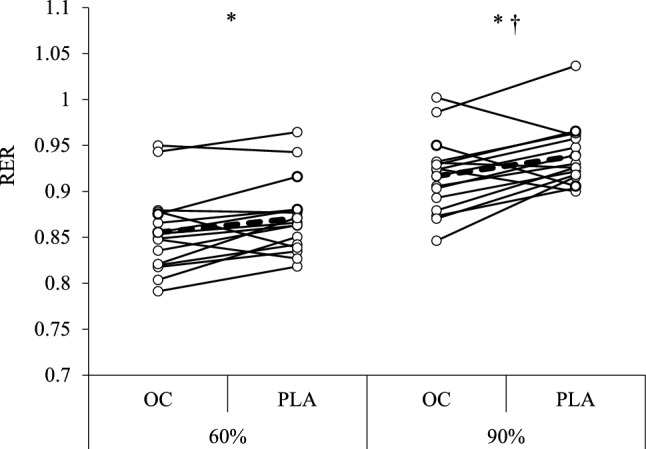
Univariate scatter plot with individual and mean respiratory exchange ratio (RER) for Oral Contraceptive (OC) and Placebo (PLA) conditions at 70% and 90% lactate threshold. * indicates a significant difference between OC and PLA and † indicates a significant difference compared to 70%LT

The aim of this study was to assess whether the acute ingestion of a combined OC affected cognition, substrate metabolism and exercise performance, compared to the ingestion of a placebo. In agreement with the primary hypotheses, RER was lower, and verbal fluency was superior following OC ingestion compared to PLA, however, there was no difference in verbal memory performance between the conditions. There was also no difference in exercise performance or VO_2_ peak between conditions, however, felt arousal and feeling scale were lower in the OC condition (Fig. 2).

The lower RER observed during sub-maximal exercise following OC ingestion compared to a placebo is similar to that observed in studies of the menstrual cycle, whereby RER was lower in the luteal phase compared to the early (Zderic et al. 2001) and mid-follicular phase (Hackney et al. 1994; Wenz et al. 1997). A lower RER is indicative of a greater proportional use of fat as a fuel, which is in line with the proposed mechanisms of oestrogen on substrate metabolism. Oestrogen influences substrate metabolism indirectly via actions on growth hormone and insulin (D’Eon & Braun 2002) and directly via glucose transporter-4 (GLUT4) translocation (Campello et al. 2017; Yue et al. 2020) and increased lipoprotein lipase and hormone-sensitive lipase activity (Campbell & Febbraio 2001; D’Eon et al. 2005) among other mechanisms (for review see Oosthuyse et al. 2023). It has been hypothesised that the oral delivery of combined OCs results in high hepatic exposure via the portal vein which could exacerbate the effects of these hormones in hepatocytes (Magkos et al. 2022) thereby resulting in marked changes to substrate metabolism. Increased oestrogen receptor-mediated 5’AMP-activated (AMPK) phosphorylation activity occurs within 10 min of oestrogen administration (D’Eon et al. 2008; Gorres et al. 2011) demonstrating that the changes in metabolism observed with the transient spike in exogenous hormones in the current study are plausible.

In contrast to some studies of sex or menstrual cycle effects on substrate metabolism during exercise (e.g., Wenz et al. 1997), the current study did not find significant differences between conditions for either fat or carbohydrate oxidation rate, despite significant differences in RER. However, as RER is a ratio of two correlated values (VO_2,_ VCO_2_), the pooled coefficient of variation was much smaller (4.9%) compared to carbohydrate (38.09%) and fat (36.3%) oxidation rates, thereby improving the likelihood of statistical significance, a known issue with ratio data (Jasienski & Bazzaz 1999). The absence of significant differences in fat and carbohydrate oxidation between the OC and placebo condition may also be related to type and timing of reproductive hormone concentrations used in the current study. Prior research has shown that the change in fat oxidation from the early follicular phase to the mid-luteal phase was positively correlated with the change in oestrogen to progesterone ratio (Hackney et al. 2022), potentially as a result of the anti-oestrogenic effect of progesterone (Hatta et al. 1988). Specifically, progesterone has been shown to repress oestrogen-mediated GLUT4 translocation (Campbell & Febbraio 2002). Participants in the current study all used the same type and concentration of progestin (150 mg Levonorgestrel) which might elicit different progestogenic effects compared to endogenous progesterone or other types (*i.e.,* generations) and concentrations of progestins in other hormone therapies (Mitchell & Welling 2020). Furthermore, 30 µg ethinyl oestradiol was used by all participants which might have different physiological effects compared to other types of oestrogen (e.g., oestradiol or oestradiol valerate) or doses of ethinyloestradiol used in many hormonal contraceptives (20–50 µg; Stanczyk et al. 2013). Further work is needed to explore the pharmacodynamic effect of different concentrations and types of hormonal contraceptives (e.g. progestin-only contraceptives), in addition to other hormonal therapies such as hormone replacement therapy (HRT).

Concomitant with a greater proportional use of fat as a fuel in the OC condition, was a lower felt arousal and feeling scale score, which are considered together as measures of affective valence (Bastos et al. 2023). Reduced affective valence in the presence of higher oestrogen concentrations has previously been observed when exercising in the luteal phase of the menstrual cycle (Prado et al. 2021) although the mechanisms behind this are unclear. Glucose availability is positively associated with affective valence (Backhouse et al. 2007; Lee et al. 2018) so it may be that lower proportional carbohydrate oxidation resulted in lower affective valence. Alternatively, OC use blunts the cortisol response to exercise (Kirschbaum et al. 1996) and down-regulates IGF-1 concentrations which mediate the positive effects of exercise on mood in females (Munive et al. 2016). Oestrogen may also directly attenuate arousal via cortical-sub-cortical control within hypothalamic–pituitary–adrenal circuitry (Goldstein et al. 2005). Typically, lower affective valences are observed with greater exercise intensity (Kilpatrick et al. 2007) or perceived exertion (Farias-Junior et al. 2020), however, treadmill speed was identical between conditions and no significant differences in RPE were observed during the sub-maximal exercise stages. Experiencing positive affective responses to exercise makes individuals more likely to continue to undertake exercise (Rhodes & Kates 2015) and, therefore, the timing of OC consumption around exercise should be researched in relation to enjoyment and participation.

Perceived exertion regulates the termination of exercise (Marcora & Staiano 2010) and, in line with the similar RPE scores during sub-maximal exercise, there were no significant differences between the OC and placebo conditions for time to exhaustion in the ramp test, VO_2peak_ or peak heart rate. It is purported that sex-hormone-related differences are more likely to impact exercise performance in longer-duration exercise, whereby glycogen sparring via increased fat oxidation may confer a performance advantage (Tiller et al. 2021). Therefore, future research exploring the effects of OC consumption timing on exercise performance should include longer-duration exercise.

For the cognitive data, verbal fluency was superior in the OC condition compared to the placebo which is in line with prior research showing improved performance in women compared to men, and improved performance in the late follicular phase and mid-luteal phases of the menstrual cycle compared to the early follicular phase (Luine 2014; Maki et al. 2002; Rosenberg & Park 2002; Solís-Ortiz & Corsi-Cabrera 2008). Griksiene and Ruksenas (2011) showed that participants using OCs containing third-generation progestins (*e.g.,* gestodene, desogestrel and norgestimate) had poorer verbal fluency compared to newer, less androgenic OCs (e.g., drospirenone). The current study used 2nd generation OCs (150 mg levonorgestrel) which are similar in androgenicity to 3rd generation OCs (Louw-du Toit et al. 2017) and, therefore, the pharmacodynamic effects observed in the current study may be different compared to other OC formulations, especially considering levonorgestrel is one of few progestins which are oestrogen-receptor alpha (ERα) agonists (Low-du Toit et al. 2017). The current study found no effect of acute OC ingestion on verbal memory, despite this often being affected by alterations to reproductive hormone concentrations such as with the menstrual cycle or oral contraceptive use (Luine 2014; Warren et al. 2014; Maki et al. 2002; Rosenberg & Park 2002) Further work is needed to explore whether this type of exogenous hormonal manipulaion uniquely improves verbal fluency and how this is mediated, in addition to exploring other OC formulations and other exogenous hormone models.

### Limitations

The study protocol included the use of a computer-based cognitive test battery comprised of the mental rotation test, Stroop task, Corsi blocks test and RVIP test. However, due to a technical issue with the software, we only had complete data sets for 9 participants and, therefore, have removed these data from the study as these data were underpowered. Further research would benefit from studying the acute effects of OC ingestion on a wider range of cognitive tasks, especially the mental rotation test which is often affected by the reproductive hormone milieu (Peragine et al. 2020). We also collected blood samples for assessment of serum ethinyloestradiol and levonorgestrel, however, a storage issue meant that these data could not be used. Future research employing this design could collect serum samples to compare the pharmacokinetic profiles of the OCs between participants and examine whether this influences the pharmacodynamic response. Whilst an a priori power analysis was conducted for primary variables of interest (RER and verbal memory), we did not conduct power calculations for secondary variables and, therefore, some inferences in the current study may be underpowered. Whilst our study found significant effects of OC consumption time on RER, arousal, affect and verbal fluency, these were moderate effect sizes (D = 0.37–0.59), similar to those seen in RER across the menstrual cycle (Hackney et al. 1995; 0.46–0.59) and further research is required to support or refute these findings. Lastly, we only included OC formulations with 30 µg ethinyloestradiol and 150 mg levonorgestrel and, therefore, further research is required to explore other OC types (e.g., progesterone-only) formulations (i.e., different progestins) and concentrations, in addition to other exogenous reproductive hormone models such as hormone replacement therapy in menopausal women.

### Implications of research

This study has shown that the acute ingestion of OCs affects substrate metabolism, affective responses to exercise and verbal fluency and, therefore, OC users could potentially manipulate the timing their OC ingestion to optimise outcomes according to their needs. Whilst additional research is required to corroborate these findings and identify other factors that may be affected by the timing of OC ingestion (*e.g.,* injury risk, thermoregulation, muscle function), this could impact how OCs are used in sport, exercise or professional settings where ergogenic effects are sought, without impacting contraceptive efficacity (Zapata et al. 2013). Importantly, the current study has implications for existing research that has been conducted on OC users as very little research has standardised the timing of OC ingestion in relation to measurements. If studies are looking to compare OC users to non-users or compare the phases of OC use, studies that do not consider the timing of ingestion of OC in relation to measurements may introduce a source of variability. The timing of OC ingestion should, therefore, be considered as a factor that could be controlled or considered in research as with other factors (e.g., Merrell et al. 2024), and evaluations of extant research in OCs users should be cautious of whether OC ingestion timing was controlled and the potential effects on research quality.

## Conclusion

This is the first study to show that physiological function is altered in the period (~ 60–120 min) following OC ingestion, at a time when exogenous reproductive hormones are known to be transiently elevated (Kuhnz et al. 1992). OC ingestion was associated with improved verbal fluency, a lower RER during exercise, and lower affective valence compared to ingestion of a placebo. Therefore, the timing of OC ingestion should be considered as a factor that may affect physical responses at rest and during exercise and further research is needed to explore this phenomenon.

## Data Availability

Data are available at 10.5281/zenodo.13144079. Registered on Open Science Framework https://osf.io/m9jr3

## Abbreviations

AMPK: AMP-activated protein kinase
ANOVA: Analysis of variance
GLUT4: Glucose transporter 4
HR: Heart rate
LT: Lactate threshold
OC: Oral contraceptive
PLA: Placebo
RAVLT: Rey auditory verbal learning test
RER: Respiratory exchange ratio
RVIP: Rapid visual information processing
RPE: Rating of perceived exertion
TTE: Time to exhaustion
VO_2_peak: Peak oxygen uptake
